# The Ubiquitin Proteasome System in Neuromuscular Disorders: Moving Beyond Movement

**DOI:** 10.3390/ijms21176429

**Published:** 2020-09-03

**Authors:** Sara Bachiller, Isabel M. Alonso-Bellido, Luis Miguel Real, Eva María Pérez-Villegas, José Luis Venero, Tomas Deierborg, José Ángel Armengol, Rocío Ruiz

**Affiliations:** 1 Experimental Neuroinflammation Laboratory, Department of Experimental Medical Science, Lund University, Sölvegatan 19, 221 84 Lund, Sweden; tomas.deierborg@med.lu.se; 2 Departamento de Bioquímica y Biología Molecular, Facultad de Farmacia, Universidad de Sevilla/Instituto de Biomedicina de Sevilla-Hospital Universitario Virgen del Rocío/CSIC/Universidad de Sevilla, 41012 Sevilla, Spain; isaalobel@gmail.com (I.M.A.-B.); jlvenero@us.es (J.L.V.); rruizlaza@us.es (R.R.); 3 Unidad Clínica de Enfermedades Infecciosas, Hospital Universitario de Valme, 41014 Sevilla, Spain; lmreal67b@gmail.com; 4 Departamento de Especialidades Quirúrgicas, Bioquímica e Inmunología, Facultad de Medicina, 29071 Universidad de Málaga, Spain; 5 Departamento de Fisiología, Anatomía y Biología Celular, Universidad Pablo de Olavide, 41013 Sevilla, Spain; empervil@upo.es (E.M.P.-V.); jaarmbut@upo.es (J.Á.A.)

**Keywords:** ubiquitin, proteasome, UPS, neuromuscular junction, synapse, neuromuscular disorder

## Abstract

Neuromuscular disorders (NMDs) affect 1 in 3000 people worldwide. There are more than 150 different types of NMDs, where the common feature is the loss of muscle strength. These disorders are classified according to their neuroanatomical location, as motor neuron diseases, peripheral nerve diseases, neuromuscular junction diseases, and muscle diseases. Over the years, numerous studies have pointed to protein homeostasis as a crucial factor in the development of these fatal diseases. The ubiquitin–proteasome system (UPS) plays a fundamental role in maintaining protein homeostasis, being involved in protein degradation, among other cellular functions. Through a cascade of enzymatic reactions, proteins are ubiquitinated, tagged, and translocated to the proteasome to be degraded. Within the ubiquitin system, we can find three main groups of enzymes: E1 (ubiquitin-activating enzymes), E2 (ubiquitin-conjugating enzymes), and E3 (ubiquitin–protein ligases). Only the ubiquitinated proteins with specific chain linkages (such as K48) will be degraded by the UPS. In this review, we describe the relevance of this system in NMDs, summarizing the UPS proteins that have been involved in pathological conditions and neuromuscular disorders, such as Spinal Muscular Atrophy (SMA), Charcot–Marie–Tooth disease (CMT), or Duchenne Muscular Dystrophy (DMD), among others. A better knowledge of the processes involved in the maintenance of proteostasis may pave the way for future progress in neuromuscular disorder studies and treatments.

## 1. Introduction

Neuromuscular disorders (NMDs) consist of a heterogeneous group of diseases, including more than 150 different types. They affect mainly the peripheral nervous system (PNS), skeletal muscle, neuromuscular junctions (NMJ), and spinal cord (SC). Although most of them have a genetic origin (and hence are inheritable), others do not have a known etiology. A common feature of them is the loss of muscle strength. Onset may occur at any stage of life, but more than 50% occur in childhood. The prevalence of the NMDs is high around the world, being about 1:3000; for instance, in Spain, approximately 60,000 cases have been reported [1]. Currently, there are no effective therapeutic treatments [1,2,3]. The majority of these neuromuscular pathologies are accompanied by protein homeostasis impairment and accumulation of misfolded proteins. Since the ubiquitin–proteasome system (UPS) plays a key role as a sentinel of protein quality, this system is a very interesting target for studying neurodegenerative diseases. In fact, several studies have revealed the fundamental role of the UPS on disorders (for review see [4]), including NMDs (spinal muscular atrophy (SMA), spinal and bulbar muscular atrophy (SBMA), X-spinal muscular atrophy, X-linked juvenile and adult-onset amyotrophic lateral sclerosis (ALS) [5,6,7,8,9]), and peripheral neuropathies (Charcot–Marie–Tooth (CMT) disease [10,11,12]).

In this review, we compile for the first time the importance of the UPS in neuromuscular function and its implication in pathological conditions.

### 1.1. Neuromuscular System

The voluntary movement, even the simplest one, implies a huge amount of information processing at the related structures and cells. The NMJ mainly has three components: (i) a motor neuron (MN) as a presynaptic component, with the soma located in the ventral horn of the SC; (ii) muscle fiber as a postsynaptic element; and, (iii) a Schwann cell as a support cell; altogether, these establish the tripartite synapse [13]. The connection between the MN and the muscle (endplate) is very specialized. In vertebrates, when an action potential arrives at the presynaptic terminal, sophisticated mechanisms lead to acetylcholine neurotransmitter release. This action triggers the contraction of the target muscle throughout nicotinic acetylcholine receptors (nAChRs). This refined mechanism requires a great maintaining of the proteins implicated. One of the main actors in the protein homeostasis is the proteasome. This system carries out a clearance of damaged and/or misfolded proteins, and several studies have pointed out its contribution to the synaptic homeostasis [14,15,16,17]. Furthermore, impairment in the protein degradation system has been related to neurodegenerative diseases and NMDs (for review, see [18]).

### 1.2. The Ubiquitin–Proteasome System (UPS)

**Figure 1:**
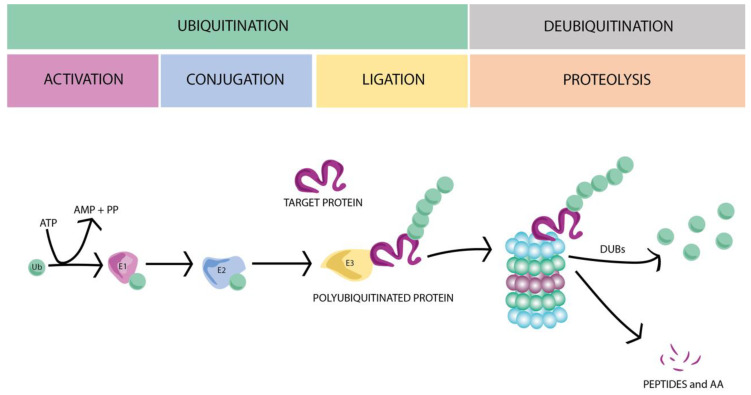
The ubiquitin–proteasome system (UPS). Ubiquitin-activating enzyme (E1) activates the ubiquitin (Ub) through an ATP-dependent reaction. Then, the activated ubiquitin is transferred to a ubiquitin-conjugating enzyme (E2) and to the target protein by an E3 ubiquitin ligase. Following several rounds of ubiquitination, the polyubiquitinated protein is recognized by the proteasome and degraded to small peptides and amino acids (AAs). To finish the UPS cycle, the polyubiquitin molecules are disassembled by deubiquitinating enzymes (DUBs) and recycled for new rounds of ubiquitination.

UPS controls and assists the protein degradation process. The ligation of ubiquitin molecules to targeted proteins plays a crucial role in specific protein degradation, and therefore in its turnover. A cascade of catalytic reactions involves three main groups of enzymes that perform the protein ubiquitination, tagging, and driving to the proteasome to be subsequently degraded: E1 (ubiquitin-activating enzyme), E2 (ubiquitin-conjugating enzyme), and E3 (ubiquitin–protein ligase), respectively (for review see [14,17,19,20]). Briefly, E1 enzymes activate ubiquitin, generating an ATP-dependent chemical bond between the C-terminal glycine (G76) to the cysteine of ubiquitin, producing a high-energy thioester intermediate. Next, E2 ubiquitin-conjugating enzymes take the activated ubiquitin and transfer it to the target substrate by an E3 ubiquitin ligase (Figure 1).

Within each group of UPS enzymes, different variants have been described: 26 for E1, over 105 of E2, more than 1003 for E3 enzymes, and 6647 deubiquitinating enzymes (DUBs) [21]. Although these numbers are higher than expected, Gao et al. [21] estimates that the number could be even bigger (738 E1s, 2937 E2s, 46,631 E3s, and 6647 DUBs from 70 eukaryotic organisms). The specificity of each substrate is partially determined by the different E2s, combined with an extensive number of different E3 enzymes. Finally, the ubiquitinated protein might be subjected to different rounds of ubiquitination, generating a polyubiquitinated substrate, which is recognized by the proteasome (26S) and degraded to small peptides and amino acids. The polyubiquitin chains are disassembled by DUBs (Figure 1), closing the ubiquitin proteasome system cycle [14,18]. The role of UPS in maintaining protein homeostasis is fundamental to correctly carry out cellular functions, such as cell cycle progression, apoptosis, transcription and development, immune response, DNA repair, and membrane transport [22,23]. UPS is also involved in the presynaptic neurotransmitter release, as shown by Rinetti and Schweizer [24]. These authors showed that the inhibition of E1 enzymes in hippocampal cultured neurons alters the synaptic frequency, suggesting the participation of ubiquitin dynamics in the regulation of synaptic transmission.

The wide function of UPS and its key role as a guard of protein quality, make the UPS a very interesting target for studying the neurodegenerative diseases that arise with the accumulation of misfolded proteins.

### 1.3. UPS and Neuromuscular System

Within the different components at the NMJ, coordinated mechanisms between the UPS and the autophagy–lysosomal degradation system are necessary to keep protein homeostasis [14,15,16,17,25,26]. In the motor neuron soma, UPS is involved in the maintaining of the cytoskeleton and regulating motor neuron differentiation [27]. In the presynaptic terminal, proteasome-related ubiquitin chains (K11 and K48) act as signals for the assembly of presynaptic terminals [28], and its inhibition prevents decreases in the size of the readily releasable pool of vesicles, accompanied by a decreased synaptic protein expression of Munc13-1 and Rim1 [29]. Moreover, the presynaptic protein Bassoon, together with the E3 ubiquitin ligase PRKN/Parkin, can also control presynaptic autophagy and proteostasis [30]. In the Schwann cells, UPS plays a role in the phenotype changes of these cells after an insult, such as nerve injury, a crucial process in myelin degeneration [31]. In muscle, UPS controls the muscle mass involving the E3 ubiquitin ligases MuRF1 and MaFbx. MuRF1 creates a complex with E2 enzymes, which targets sarcomeric proteins for degradation [32]. One of these E2 enzymes (E2E1) has a protective effect on skeletal muscles of atrophy mice, and has been described as a fiber-specific in slow-twitch muscle fibers (type I and type IIA) [33]. Therefore, alterations in the protein degradation system, autophagy processes, and lipid metabolism have been related to neurodegenerative diseases and NMDs [18,34,35].

In Table 1, we summarize the main ubiquitin system protein alterations associated with motor function impairments and NMDs.

## 2. UPS in Neuromuscular Disorders (NMDs)

The classification of the NMDs is a complex problem, due to the variation in the specific hallmarks of the diseases. According to its anatomical location, researchers distinguish four main groups (for review, see [1,3]): (i) MN diseases located in the anterior horn cells at the SC, (ii) peripheral nerve diseases (PNs), (iii) NMJ diseases, and (iv) muscle diseases. It is necessary to clarify that some of the diseases explained in this review could be included in more than one of the groups mentioned above, such as ALS or SMA. Although SMA and ALS are characterized by the loss of motor neurons, alterations in the NMJ are also present. In this review, we will be focused on the representative diseases of each group, paying special attention to the UPS implication.

### 2.1. Motor Neuron Diseases

The motor unit, first established by Liddell and Sherrington in 1925 [82], is constituted by MNs and their innervated muscle fibers. Each MN can innervate between 2 and 2000 muscle fibers, being on average 100 fibers per motor unit [83]. Alpha MNs are located in the anterior horns of the SC and in the motor nuclei of the cranial nerves, and their axons connect the muscles. The progression of the MN diseases consists in a loss of control of the skeletal muscle activity. Therefore, usual tasks, such as walking, breathing, speaking, and swallowing are impaired. This group includes diseases like ALS, progressive bulbar palsy, primary lateral sclerosis, progressive muscular atrophy, SMA, Kennedy’s disease, and post-polio syndrome (National Institutes of Health (NIH) publication no. 19-NS-5371). In this review, we will explain the two more frequent diseases (SMA and ALS) and their relation with the UPS.

#### 2.1.1. Spinal Muscular Atrophy (SMA)

SMA is a progressive degenerative disease affecting lower MNs. The clinical consequence of the loss of MNs is weakness and atrophy of muscles, accompanied by an impairment of neurotransmission in the NMJ [84], being the most common genetic cause of death in childhood [85]. In addition, SMA patients present an increased susceptibility to develop dyslipidemia and liver steatosis [86]. In ~95% of cases, the disease is due to mutations in the *SMN1* gene leading to reduced levels of the ubiquitously expressed, full-length survival of motorneuron (SMN) proteins [87]. The severity of the disease is inversely associated to the number of copies of the *SMN2* gene, which produces mainly SMN without exon 7 (SMNΔ7), and a reduced amount of full-length SMNs. The SMNΔ7 is polyubiquitinated and rapidly degraded by the UPS, retaining only ~10% of full-length SMN as functional protein [88]. In fact, increasing the expression of *SMN2* alleviates the MN lost in a mouse model of SMA [89]. The SMN protein is known to play key roles in several core canonical cellular pathways, including snRNP biogenesis and pre-mRNA splicing [90], actin dynamics [91,92], phosphatase and tensin homolog–mediated (PTEN-mediated) protein synthesis pathways [93], and translational regulation [94]. Furthermore, functional analysis of synaptosomes from the SMA mouse model *Smn^2B/−^* revealed a significant increase in proteins involved in the cholesterol biogenesis and mitochondrial dynamics [95].

SMN is known to be monoubiquitinated by means of several components of the ubiquitin pathway (reviewed in [96]). This protein modification could be implicated in MN-specific functions of SMN, instead of its degradation, including protein trafficking and intracellular localization [97]. Since the disease is associated with low levels of the SMN protein in most cases, its homeostasis is essential for the normal functioning of the MN [96]. Multiple facts point out the involvement of UPS in this disease: (i) inhibition of the proteasome in SMA-patient-derived fibroblasts increases the intracellular abundance of SMN [98]; (ii) pharmacological inhibition of ubiquitination of SMN has increased protein levels and slowed disease progression of severe SMA in a mouse model [99]; (iii) Kwon et al. described the E3 ubiquitin ligase mind bomb 1 (Mib1), which interacts and ubiquitinates SMN and facilitates its degradation—knocking down the Mib1 orthologue improved neuromuscular function in *C. elegans*-deficient SMN [64]; (iv) in an SMA mouse model, Wishart et al., [100] showed an important role of ubiquitin homeostasis, mediated mainly by modifications in levels of ubiquitin-like modifier-activating enzyme 1 (UBA1); (v) mutations in UBA1 have been found in the X-linked infantile SMA [6,7]; and (vi) ubiquitin-specific protease 9x (Usp9x) deubiquitinates and stabilizes SMN, but it does not regulate the ubiquitination and stability of SMNΔ7, making it more susceptible to proteasome degradation than SMN [101].

#### 2.1.2. Amyotrophic Lateral Sclerosis (ALS)

ALS is a progressive, invariably fatal disease characterized by the death of upper and lower MNs leading to progressive paralysis, atrophy of denervated muscles, and severe disability. Patients typically die within 5 years of onset as a consequence of progressive restrictive respiratory failure [102,103]. Moreover, sexual dimorphism has been revealed in the clinical manifestation, appearing later in women than in men and with a higher survival rate, which seems to be associated with a lower accumulation of Ub-K48 and therefore, a higher capacity of the proteasome in the spinal cord [104]. A common hallmark in ALS is the increase of misfolded proteins into the damaged neurons, leading to the formation of soma inclusions that are frequently tagged with ubiquitin [65]. In addition, autophagy and UPS alterations have been described in the NMJ modifications found in early ALS [25]. Unlike SMA, ALS has a sporadic origin in most patients; in fact, only 10–15% of cases are presented as familial forms. ALS can be mainly considered as a multifactorial disease, since studies in animal models and in human postmortem samples have shown alterations in multiple essential cellular functions, such as control of oxidative stress and mitochondrial function, glutamate excitotoxicity, neuroinflammation, and cytoskeleton alterations (to review, see [105,106,107,108]). In addition, these alterations are usually accompanied by protein accumulation and proteostasis impairment. Moreover, recent genome-wide association studies have found genes whose functions are related to the aforementioned cellular processes associated with this disease [109].

The different genetic mutations linked to ALS are associated with the presence of aberrant protein aggregates and inclusions in MNs, which lead to the accumulation and consequent overload and dysfunction of the UPS (for review, see [65]). This is related to loss of function of proteins essential for the survival of MNs, the gain of neurotoxic function, or a combination of both [110]. The first reported mutation associated with familial ALS was the gene encoding copper–zinc superoxide dismutase 1 (SOD1), an essential enzyme in the antioxidant defense of cells [111]. This mutation leads to increased oxidation of proteins, which in turn can undergo aberrant structural and functional changes. Other mutations include E3 ligases (such as Dorfin [112], CHIP [113], Gp78 [114], Trapd associated NEDL1 [115], and MITOL [116]) that are activated in MNs and interact with SOD1 to remove the mutated, SOD1-rich aggregates. UPS alteration has also been described in cells expressing mutant Ubiquilin-2 (UBQLN2), and inclusions of this protein have been found in a wide spectrum of ALS patients [5]. The UBQLN2 mutation disrupts the traffic between the endoplasmic reticulum and Golgi apparatus, and as a consequence, creates an inefficient secretory system to maintain viable synaptic functions, causing a disruption of motor neuron functions [117]. Another aspect presumably associated with UPS deterioration is the delocalization and accumulation of TDP-43 aggregates in the cytoplasm in ALS cases [118]. TDP-43 is a common hallmark in cytoplasmic aggregates, found in ~97% of ALS patients’ post-mortem tissue [119]. Therefore, different research approaches have been focused on the protein aggregate formation and its consequences in normal cellular processes that lead to ALS pathology. Watabe et al. [70] have identified the E3 ubiquitin ligase Praja 1 (PJA1), which is capable of preventing the formation of neuronal TDP-43 aggregates, and therefore, it has been postulated as a potential therapeutic target for ALS. Nevertheless, as with other protein aggregation diseases, where protein aggregation is a late feature of the pathology development, the benefit of using TDP-43 aggregates as a therapeutic target could be relative [120]. Another potential biomarker recently found is the binding of the chromosomal region protein of Smith–Magenis syndrome, candidate 8 (SMCR8) to C9orf72, one of the most implicated proteins in ALS [121].

### 2.2. Peripheral Nerve Diseases

The motor and sensory neurons are located in the spinal cord, and motor nuclei, along with their axons, form the PNS. The axons connect the neurons with their distant target and transmit the information to perform the required action. Axons are grouped together in spatially arranged motor or sensory bundles called fascicles [122]. There are abundant types of peripheral neuropathies, but in this review, we will be focused on CMT disease and Friedreich ataxia (FRDA). Both diseases are accompanied by aberrant protein accumulation, and therefore, loss of protein homeostasis.

#### 2.2.1. Charcot–Marie–Tooth Disease (CMT)

CMT disease is an inherited, dominant, peripheral neuropathy that affects the PNS with demyelinating processes [123]. This disease is characterized by progressive muscle weakness and atrophy in combination with sensory difficulties [124]. They can be subdivided into two main types of peripheral neuropathies. Here, we will describe more in detail type 1, because it is the most common form of CMT. Specifically, we will be focused on the subtype 1A (CMT1A), caused by duplication or point mutations in the peripheral myelin protein 22 (PMP22) [125].

PMP22 is a transmembrane protein expressed in the peripheral nerves that participate in the myelin structure, as well as in the Schwann cells, where PMP22 is involved in their differentiation and migration [126]. In fact, its absence alters cholesterol metabolism in the Schwann cells, leading to myelinization deficits [127]. In normal cells from animal models, the majority of the newly synthesized Pmp22 protein is turned over by the proteasome [128]. Aberrant expression of Pmp22 induces protein aggregation that cannot be degraded by the UPS [129]. In fact, an increase of PMP22 aggregates in the cytoplasm of Schwann cells were found in patients with CMT1A [130], suggesting that the UPS may contribute to the pathogenesis of the disease. Studies in a Trembler J mouse model of CMT neuropathy have described a slowed turnover of Pmp22 protein, which leads to its accumulation along with a reduction in the proteasomal function in nerves [129]. Furthermore, in this mouse model, the enhancement of the proteasome function improves the processing of Pmp22 and the myelination capacity of the Schwann cells [131]. The presence of the mutation in Pmp22 occurs starting with the CMT1A patients’ birth. However, they only exhibit disease symptoms later in life. Therefore, the instability of PMP22 protein must be accompanied by a combination of both toxicity of the misfolded proteins and the formation of aggresomes and loss of the native function, leading to detrimental autophagy processes with age [132].

#### 2.2.2. Friedreich Ataxia (FRDA)

FRDA is a genetic neurodegenerative disorder that affects mainly the CNS and PNS, the muscle-skeletal system, the cardiovascular system, and the endocrine pancreas. It is the most commonly inherited ataxia, and progressively impairs movement coordination, gait instability, muscle weakness, and sensory loss [133,134]. It is caused by guanine–adenine–adenine (GAA) repeat hyper- expansion within the first intron of the frataxin (*FXN*) gene, on the chromosome 9q21.11, which results in reduced transcription of the gene [135]. In FRDA patients, the GAA triplets can reach 66 to more than 1700 repetitions, compared with 10 to 35 found in healthy humans [136], and the length of this repetitions has been inversely correlated with the severity and the age of onset of the disease [137].

Frataxin is a mitochondrial protein that is highly conserved, with an important function in iron metabolism [138,139]. Therefore, defects in this protein reduced mitochondrial respiration, increased sensitivity to oxidative stress, and induced lipid accumulation, leading to neuronal degeneration, predominantly in the dorsal root ganglia [139,140,141,142,143]. Prior to being imported to the mitochondria, frataxin is synthesized as a precursor in the cytosol, which is processed in a two-step catalytic process, generating a mature form by mitochondrial peptidases [144,145]. To date, there is no treatment for this devastating disorder, and the finding of the mechanisms involved in the frataxin processing could help in the design of a potential therapeutic strategy. Actually, studies in animal models of FRDA show that the restoration of Fxn levels improves the motor symptoms [133].

In 2011, Rufini et al. reported that during the normal maturation of FXN, a significant fraction of the precursor was targeted for ubiquitin proteasome degradation [146]. Specifically, they found that a single lysine residue in the position 147 (K^147^) is the main target of ubiquitination through the UPS pathway. Moreover, using HeLa cell cultures co-infected with frataxin mutant protein lacking the K^147^, they showed an increase in the stability and prolonged half-life of cultured cells [134,146]. In the same way, Nabhan and colleagues showed that whereas the mature form of frataxin is resistant to change, the precursor levels fluctuate in cell cultures, as well as in patient-derived or control lymphoblasts [147]. Thus, controlling the UPS could be a promising approach to increase the frataxin levels. Regarding this, in 2017 Benini et al. identified a RING E3 ligase RNF126 as a specific enzyme that participates in the ubiquitination of frataxin for degradation [69]. Interestingly, knockdown of this E3 ligase in FRDA-derived fibroblasts induces frataxin accumulation, suggesting that this protein might be playing a role in the FRDA pathology, thus pointing it out as a new beneficial tool for FRDA treatment.

### 2.3. Neuromuscular Junction

As we mentioned above, the NMJ is the synaptic contact between the MN and the muscle. It is a chemical synapse where the neurotransmitter (acetylcholine in mammals) is released by the presynaptic terminal (MN) towards the synaptic cleft, where it will interact with the nicotinic receptors located in the postsynaptic motor end-plate. The entry of cations into the muscle fiber starts a depolarization (evoked end-plate potential), which in turn will generate the opening of the sodium voltage-dependent channels, triggering the contraction process in a tightly regulated manner. Among these regulated mechanisms, the turnover of the implicated proteins needs to be controlled, and consequently, all proteins must be ready to use for each round of neurotransmitter release. Several studies have revealed the fundamental role of the neuronal UPS on homeostasis maintenance at neuromuscular synapses [16,148,149]. Therefore, the impairment of the UPS-mediated protein homeostasis in neuromuscular synapses triggers motor diseases. Thus, (i) the impairment of anaphase-promoting complex (Apc), an E3 ubiquitin ligase, at the fly NMJ restricts the number of presynaptic boutons [39]; (ii) overexpression of PDZRN3, an E3 ubiquitin ligase in skeletal muscle, leads to defects in growth and maturation of the NMJ [15]; (iii) deficiency of E3 ubiquitin ligase cullin-3 has been reported in the nemaline myopathy, characterized by the presence of protein aggregates like non-muscular α-actinin ACTN1 in the myofibers as a consequence of an inefficient degradation by the UPS altering the normal development of NMJ [56]; (iv) loss of function of the E3 ubiquitin ligase gigaxonin, which plays an important role in neurofilament architecture, causes giant axonal neuropathy, impairing MN specification and somitogenesis and suppressing NMJ formation and locomotion [57]; (v) in 2015, our group showed that the spontaneous mutation in Herc1 E3 ubiquitin ligase induces an impairment in the evoked neurotransmitter release at the NMJ, and this phenotype appears previously to the ataxic phenotype characteristic of this mouse model called *tambaleante* [42]; (vi) the loss of the ubiquitin carboxyl-terminal hydrolase 14 (Usp14) generates an impaired proteasome function at the synapses, causing widespread changes in the endplates and developmental anomalies at the NMJ in mice model [79]; and (vii) overexpression of Drosophila eye fat facets (Faf), a deubiquitinating enzyme, in the developing nervous system is associated with effects on synaptic transmission at the NMJ [76].

In spite of this, in some diseases, such as myasthenia gravis (to review, see [150]), it is not clear if the proteosomal dysfunction is a cause or consequence of the pathology leading to the disease.

### 2.4. Muscle

The muscle is a contractile tissue, and the active part of the locomotor system has an essential role as the protein reservoir of the body. Protein homeostasis is also essential for its normal function.

#### 2.4.1. Duchenne Muscular Dystrophy (DMD)

DMD is an inherited, X-linked disease characterized by an alteration of gait, progressive muscle weakness and wasting, and variable degrees of cognitive impairment [151,152,153]. Nonsense mutations or out-of-frame deletions in the dystrophin gene (*DMD*) result in the lack of dystrophin protein that causes the severe phenotype of DMD [154]. Dystrophin is an important structural component of both skeletal and cardiac muscles. It binds the cytoskeletal protein actin to the dystrophin–glycoprotein complex (DGC) on the cell membrane, which is important in the stabilization of muscle cell membranes during muscle contractions [155]. Dystrophin deficiency induces membrane instability, leading to the activation of molecular pathways to maintain protein homeostasis, including the UPS. In fact, abnormal increases in both proteasome and ubiquitin levels have been found in the cytoplasm of necrotic fibers from DMD human muscle biopsies [156]. In addition, glucocorticoids are a common treatment used in DMD; however, in 2019, Hammers et al. [157] reported that chronic treatment with prednisolone causes muscle wasting and activates UPS degradation, as well as inhibition of the muscle protein synthesis, in a mouse model of DMD. Therefore, the inhibition of the proteasome has been considered as a promising therapeutic approach in DMD, as supported by experimental results using the *mdx* mouse model [158], *C.elegans* [67], and golden retriever muscular dystrophy (GRMD) dog model [66]. The treatment with the proteasome inhibitor MG132 and the gene deletion of Chn-1/CHIP decelerates the progression of muscular dystrophy in *C.elegans* [67]. In *mdx* mice, proteasome inhibition, using MG132 or Velcade, promoted a reduction of membrane damage in the myofibers and ameliorated the histopathological markers of the DMD. However, treatment with proteasome inhibitors in skeletal muscle biopsies from DMD patients did not show a phenotype rescue [60,158,159]. Similarly, specific inhibitors of the ubiquitin-conjugating enzymes have been studied in DMD progression. In a new *mdx* mouse model carrying the human mutation L54R for dystrophin gene -cultured myoblasts [160] revealed four E3 ubiquitin-ligases (Zfand5, FBXO33, Amn1, and Trim75) that might impact the missense dystrophin levels. In humans, dystrophy deficiency displays a selective induction of the ubiquitin ligase TRIM32, which is selectively expressed in the satellite cells and regenerative areas in the skeletal muscle [60].

#### 2.4.2. Other Muscular Dystrophies

There are other muscular disorders where the loss of protein homeostasis is involved. An example of this is the myotonic dystrophy type1 (MD). This disease is characterized by progressive myopathy and multiorgan failure, caused by an unstable expansion of non-coding CTG repeats of the DM1 protein kinase (*DMPK*) gene [161]. Vignaud et al. described defective UPS activity in the muscles from transgenic mice DM1, with overexpression of Fbx032 or Murf1, both belonging to E3 ubiquitin ligase family [162]. Another example is facioscapulohumeral muscular dystrophy (FSHMD), associated with aberrant expression of the full-length isoform of DUX4 (DUX4-FL) [163]. Using human myogenic cell cultures, Homa et al. found that the DUX4-FL isoform impairs protein turnover, mimicking the effect of MG132 and inducing TDP-43 aggregation [164]. Therefore, the restoration of proteostasis emerges as a promising approach to find an effective treatment for these disorders.

## 3. Future Directions

In carrying out this review, we have been able to verify that the majority of neuromuscular diseases are accompanied by an imbalance in protein homeostasis. This is not unique for NMDs, but is also present in the vast majority of other neurodegenerative diseases. Therefore, a greater knowledge of the processes involved in the maintenance of proteostasis is required. This will be essential to advancing knowledge of the disease mechanisms of these disorders, and thus, a common effort should be made to search for new targets for the treatment of these diseases, the majority being incurable so far.

## Figures and Tables

**Table 1:** Direct protein alterations in the ubiquitin system that induce motor system disorders.

Proteins	Organism	Function and Molecular Consequences Linked to the Dysregulation	Neuromuscular disorders and Motor Impairments	Refs.
**Ubiquitin-Activating Enzymes (E1_S_)**				
Uba1	*Mus musculus*	Modulation of ubiquitin homeostasis and regulation of sensory motor connectivity. Its mutation causes the degeneration of lower motor neurons in the anterior horn of the spinal cord.	Spinal muscular atrophy (SMA) and X-linked SMA	[36,37]
**Ubiquitin-Conjugating Enzymes (E2_S_)**				
Ubc-25	*C. elegans*	Maintenance of neuromuscular functions. The loss of function induces uncoordinated mode of locomotion.	Severe signs of a progressive paralysis	[38]
E2e1	*Mus Musculus*	Control of contractile and metabolic properties of the skeletal muscles through interaction with MuRF1 and telethonin. Its knockdown aggravates the atrophying process in Dex-treated mice.	Muscle atrophy	[33]
**Ubiquitin Ligases (E3_S_)**				
Apc	*C. elegans* *D. melanogaster*	Regulation of axonal morphogenesis, synaptic size, and activity. The loss of function increases muscle excitation at the neuromuscular junction (NMJ).	Convulsions	[39,40]
Herc1	*Mus musculus*	Important in motor function, neuromuscular transmission and peripheral myelination. Gly483Glu substitution induces protein overexpression that alters the NMJ structure.	Severe ataxia, uncoordinated gait, irregular hindlimb posture, and trembling. Decreased synaptic release, altered non-myelinating Schwann cells at NMJ, and anomalous myelination	[41,42]
Hiw Rpm-1 Phr1	*D. melanogaster* *C.elegans* *Mus musculus*	Presynaptic regulators of synapse formation and growth. The loss of function increases the number of NMJ boutons, organizes the presynaptic terminals at GABAergic NMJs, and a sprouting of nerve terminals.	Synaptic release defects and altered NMJ development	[43,44,45,46,47,48]
Murf1	*Mus musculus*	Regulation of muscle protein degradation by the UPS. The gene deletion is involved in the resistance to atrophy.	Muscle atrophy	[49,50,51]
Mafbx	*Mus musculus*	Important in muscle maintenance. Its overexpression in myotubes is related to atrophy.	Muscle atrophy	[49]
Nedd4	*D. melanogaster* *Mus musculus*	Regulation of formation and function of the NMJ. In flies, its overexpression causes defects in backward innervation and increases the number of nerve branches. In mice, the deficient mutant show aberrant innervation patterns and structure of the nerve terminals.	Flies: Abnormal larval locomotion Mice: premature embryonic lethality	[52,53,54,55]
Pdzrn3	*Mus musculus*	Regulation of the expression of muscle-specific receptor tyrosine kinase (MuSK), an organizer of postsynaptic development at the NMJ. Its overexpression reduces MuSK expression, leading to a reduction in the NMJ size.	Defects in the growth and maturation of the neuromuscular junction	[15]
Cullin-3	*Mus musculus*	Muscle protein breakdown. Its absence is characterized by a decreased neddylation and polyubiquitylation, as well as by an accumulation of non-muscular α-actinins in muscles, altering the normal development of the NMJ.	Nemaline myopathies	[56]
Gigaxonin	*Danio rerio*	Involved in the decision of neuronal and muscular fate in vertebrates. Its repression impaired motor neuron specification and somitogenesis, and suppressed NMJ formation and locomotion	Giant axonal neuropathy	[57]
UBE3A E6-AP	*Mus musculus* *Homo sapiens* *Rattus*	Ubiquitin ligase and transcriptional coactivator. Its deletion induces delayed development of reflexes, motor deficiencies. And fine motor skills.	Angelman syndrome	[58,59]
TRIM32 TRIM75	*Homo sapiens* *Mus musculus*	Involved in the control of myogenesis. Reduced levels or abnormal functionality leads to loss of ubiquitination and accumulation of tripartite motif-containing protein (TRIM) substrates within the muscle fibers.	Altered myogenesis, premature senescence of skeletal muscle, reduced proliferation, and differentiation of myoblasts; Duchenne muscular dystrophy	[60,61,62,63]
Mib1	*C. elegans*	Interaction and ubiquitination of survival of motorneuron protein (SMN), facilitating its degradation. It induces modifications in the UBA1 levels, knocks down Mib1 orthologues, and improves neuromuscular function in an motorneuron-deficient *C. elegans* model.	Abnormal larval locomotion	[64]
Dorfin Chip Gp78 Trapd-Nedl1 Mitol	*Mus musculus*	They interact and ubiquitinate mutated SOD1 proteins, leading to their degradation. The expression of these ligases is associated to a protective function.	Amyotrophic lateral sclerosis	[65]
Mdm2	*Canis lupus familiaris*	Apoptotic inhibition by targeting p53 for degradation by the proteasome. Decreased levels in the left ventricle are related to decreased proteasome activity.	Duchenne muscular dystrophy	[66]
Chn-1/CHIP	*C.elegans*	Critical role in the ubiquitylation in the control of muscle wasting and degeneration. Its deletion decelerates the progression of muscular dystrophy.	Duchenne muscular dystrophy	[67,68]
Ubqln2	*Mus musculus*	Member of the ubiquitin-like protein family involved in proteasome degradation. Inclusions of mutant UBQLN2 appear in ALS patients.	Amyotrophic lateral sclerosis	[5]
RNF126	*Homo sapiens*	It participates in the ubiquitination of frataxin for degradation. Knockdown of RNF126 induces frataxin accumulation.	Friedreich ataxia	[69]
Praja1 (PJA1)	*Rattus*	It controls phosphorylation and proteosomal degradation of TDP-43. Possible mechanism for the prevention of ALS is linked to the ability to conjugate and prevent the formation of TDP-43 aggregates.	Amyotrophic lateral sclerosis	[70]
**Deubiquitinating Enzymes (DUBs)**				
UCH-L1	*Mus musculus* *Homo sapiens*	Processing of ubiquitin precursors and ubiquitinated proteins. Its absence impairs the synaptic transmission at the NMJ, inducing profound structural defects at the presynaptic nerve terminals and denervation of the muscles.	Gracile axonal dystrophy and neurodegeneration of the peripheral nervous system	[71,72,73,74,75]
Fat facets (faf)	*D. melanogaster*	It antagonizes ubiquitin-mediated proteolysis, preventing protein degradation and controlling synapse development. Its overexpression increases the number of synaptic boutons, re-elaborates the synaptic branching pattern, and disrupts the synaptic function.	Defects in the synaptic transmission at the neuromuscular junction	[76]
Usp14	*Mus musculus*	Crucial for synaptic development and function at NMJ. Its catalytically inactive form causes developmental deficits in the NMJ structure and synaptic transmission. Its loss causes presynaptic defects.	Severe tremors, hind limb paralysis, and postnatal lethality	[77,78,79,80,81]
